# Molecular characterization of the virulent infectious hematopoietic necrosis virus (IHNV) strain 220-90

**DOI:** 10.1186/1743-422X-7-10

**Published:** 2010-01-19

**Authors:** Arun Ammayappan, Scott E LaPatra, Vikram N Vakharia

**Affiliations:** 1 Center of Marine Biotechnology, University of Maryland Biotechnology Institute, Baltimore, 701 East Pratt Street, Baltimore, Maryland 21202-3101, USA; 2 Department of Veterinary Medicine, University of Maryland, College Park, MD 20742, USA; 3 Clear Spring Foods, Inc., Research Division, P.O. Box 712, Buhl, ID 83316, USA

## Abstract

**Background:**

Infectious hematopoietic necrosis virus (IHNV) is the type species of the genus *Novirhabdovirus*, within the family *Rhabdoviridae*, infecting several species of wild and hatchery reared salmonids. Similar to other rhabdoviruses, IHNV has a linear single-stranded, negative-sense RNA genome of approximately 11,000 nucleotides. The IHNV genome encodes six genes; the nucleocapsid, phosphoprotein, matrix protein, glycoprotein, non-virion protein and polymerase protein genes, respectively. This study describes molecular characterization of the virulent IHNV strain 220-90, belonging to the M genogroup, and its phylogenetic relationships with available sequences of IHNV isolates worldwide.

**Results:**

The complete genomic sequence of IHNV strain 220-90 was determined from the DNA of six overlapping clones obtained by RT-PCR amplification of genomic RNA. The complete genome sequence of 220-90 comprises 11,133 nucleotides (GenBank GQ413939) with the gene order of 3'-N-P-M-G-NV-L-5'. These genes are separated by conserved gene junctions, with di-nucleotide gene spacers. An additional uracil nucleotide was found at the end of the 5'-trailer region, which was not reported before in other IHNV strains. The first 15 of the 16 nucleotides at the 3'- and 5'-termini of the genome are complementary, and the first 4 nucleotides at 3'-ends of the IHNV are identical to other novirhadoviruses. Sequence homology and phylogenetic analysis of the glycoprotein genes show that 220-90 strain is 97% identical to most of the IHNV strains. Comparison of the virulent 220-90 genomic sequences with less virulent WRAC isolate shows more than 300 nucleotides changes in the genome, which doesn't allow one to speculate putative residues involved in the virulence of IHNV.

**Conclusion:**

We have molecularly characterized one of the well studied IHNV isolates, 220-90 of genogroup M, which is virulent for rainbow trout, and compared phylogenetic relationship with North American and other strains. Determination of the complete nucleotide sequence is essential for future studies on pathogenesis of IHNV using a reverse genetics approach and developing efficient control strategies.

## Background

The infectious hematopoietic necrosis virus (IHNV) is probably one of the most important fish viral pathogens causing acute, systemic and often virulent disease predominantly in both wild and cultured salmon and trout [1,2]. The first reported epidemics of IHNV occurred in sockeye salmon (*Oncorhynchus nerka*) fry at Washington and Oregon fish hatcheries during the 1950s [3-5]. IHNV is native to salmonids of the Pacific Northwest region of North America and its current geographical range extends from Alaska to northern California along the Pacific coast and inland to Idaho [1,6]. IHNV has spread to Asia and Europe, most likely due to the movement of infected fish and eggs [2].

As for all the *Rhabdoviridae*, the genome of IHNV consists of a single-stranded negative-sense RNA. The gene order of IHNV is 3'-leader-N-P-M-G-NV-L-trailer-5' [7]. The negative-strand RNA genome is connected tightly with the nucleoprotein N and forms the core structure of virion. This encapsidated genomic RNA is also associated with the phosphoprotein P and polymerase protein L, which is involved in viral protein synthesis and replication. Their genome codes for five structural proteins, a nucleoprotein (N), a polymerase-associated protein (P), a matrix protein (M), an RNA-dependent RNA polymerase (L) and a surface glycoprotein (G), and a nonstructural protein (NV).

The diversity among IHNV isolates in the Hagerman Valley region was first reported by LaPatra, who used monoclonal and polyclonal antibodies to examine the heterogeneity of serum neutralization profiles of 106 IHNV isolates at four rainbow trout culture facilities between 1990 and 1992 [8,9]. Ten different serum neutralization groups were found, with three groups representing the majority (91%) of the isolates. Later, based on partial sequence analyses of the G gene of 323 field isolates, three major genetic groups of IHNV were defined, designated as the U, M, and L genogroups [10,11]. The M genogroup is endemic in the rainbow trout farming region in Idaho where phylogenetically distinct sub-groups, designated MA-MD have been reported [12]. The MB, MC, and MD sub-groups are the three most prevalent and widely distributed types of IHNV in the virus-endemic region, and they have been shown to co-circulate in the field for over 20 years [12]. To date, the complete nucleotide sequence of low virulence WRAC strain, belonging to the MA sub-group, and strain K (Kinkelin, France) has been determined [13,14]. Isolate 220-90 of the MB sub-group is virulent to rainbow trout and widely used as a challenge virus in many studies [8-12]. However, the complete nucleotide sequence of this isolate is not available. Therefore, to find out the molecular characteristics of IHNV isolate 220-90, we analyzed the entire genomic sequences and compared it with other IHNV strains.

## Methods

### Cells and Viruses

The IHNV strain 220-90 was kindly provided by Scott LaPatra, Clear Springs Foods Inc., Idaho, USA. The virus was initially recovered from acutely infected juvenile rainbow trout during routine examinations of hatchery-reared fish, conducted from 1990 to 1992 in the Hagerman Valley, Idaho, USA [8]. Specimens for virus isolation were collected when mortality increased above 200 fish day^-1^. Viruses were isolated and identified by methods previously described [15]. The *epithelioma papulosum cyprini *(EPC) cell line from common carp *Cyprinus carpio *[16] was used for the isolation, propagation, and identification of IHNV isolates. Cells were propagated in minimum essential medium (MEM) supplemented with 10% fetal bovine serum and 2mM L-glutamine (ATCC, Manassas, VA). For routine cell propagation, the EPC cells were incubated at 28°C. To propagate the virus, the cells were infected and incubated at 14°C until cytopathic effects were complete. The supernatant was collected 5 days post-infection, clarified and stored at -80°C for further processing.

### RT-PCR amplification of the IHNV genome

Viral RNA was extracted from cell culture supernatant using Qiagen RNAeasy kit, according to manufacturer's instructions (Qiagen, Valencia, CA), and stored at -20°C. The consensus PCR primers were designed using published IHNV genome sequences (GenBank accession numbers X89213; L40883) from the National Center for Biotechnology Information (NCBI). The complete genome sequences were aligned, and highly conserved sequence segments were identified and used to design overlapping primers. The oligonucleotide primers used in this study are listed in Table 1. RT-PCR amplification of the IHNV genome was carried out essentially as described for viral hemorrhagic septicemia virus (VHSV), using Superscript III RT™ and *pfx*50™ PCR kits from Invitrogen, Carlsbad, CA [17]. The RT-PCR products were purified and cloned into a pCR2.1 TOPO^® ^TA vector from Invitrogen.

**Table 1:** Oligonucleotides used for cloning and sequencing of the IHNV genome

IHNV primers	Sequences	Position
IHNV 1F	GTATAAGAAAAGTAACTTGAC	1-21

IHNV 1R	CTTCCCTCGTATTCATCCTC	2097-2078

IHNV 2F	GCAGGATCCCAAGAGGTGAAG	2033-2053

IHNV 2R	GGAACGAGAGGATTTCTGATCC	3819-3818

IHNV 3F	CAGTGGATACGGACAGATCTC	3767-3787

IHNV 3R	CTTGGGAGCTCTCCTGACTTG	5579-5559

IHNV 4F	GTACTTCACAGATCGAGGATCG	5523-5544

IHNV 4R	CGGGGACTCTTGTTCTGGAATG	7147-7128

IHNV 5F	CGTACCAGTGGAAATACATCGG	7098-7119

IHNV 5R	CAGGTGGTGAAGTAGGTGTAG	9018-8997

IHNV 6F	GAGGGAGTTCTTTGATATTCCC	8931-8952

IHNV 6R	ATAAAAAAAGTAACAGAAGGGTTCTC	11130-11105

IHNV NheR	CGTTTCTGCTAGCTTGTTGTTGG	525-503

IHNV 1MF	ACAGAAGCTAACCAAGGCTAT	729-749

IHNV 2MF	AGATCCCAATGCAGACCTACT	2610-2630

IHNV 3MF	GTATCAGGGATCTCCATCAG	4322-4341

IHNV 4MF	GATACATAAACGCATACCACA	6113-6133

IHNV 5MF	TCAGAGATGAAGCTCAGCAA	7546-7565

IHNV 6MF	AACACCATGCAGACCATACTC	9559-9579

IHNV 5'End	CGATATTGAAGAGAAAGGAATAAC	10692-10715

Oligo (dT)	GCGGCCGCTTTTTTTTTTTTTTTTTTTTT	

To identify the 3'-terminal region of the genomic RNA, viral RNA was polyadenylated as described previously (17), and used as a template for RT-PCR amplification. The cDNA was reverse transcribed using an oligo (dT) primer (5'-GCGGCCGCTTTTTTTTTTTTTTTTTTTTT-3'), followed by PCR with the IHNV-specific primer NheR (5'- CGTTTCTGCTAGCTTGTTGTTGG-3'). The 5'-terminal of genomic RNA was identified by rapid amplification of the 5'-end, using a 5'RACE kit (Invitrogen, Carlsbad, CA), according to manufacturer's instructions.

### Sequence and phylogenetic tree analysis

Plasmid DNA from various cDNA clones was sequenced by dideoxy chain termination method, using an automated DNA sequencer (Applied Biosystems Inc., Foster City, CA). Three independent clones were sequenced for each amplicon to exclude errors that can occur from RT and PCR reactions. The assembly of contiguous sequences and multiple sequence alignments were performed with the GeneDoc software [18]. The pair-wise nucleotide identity and comparative sequence analyses were conducted using Vector NTI Advance 10 software (Invitrogen, CA) and BLAST search, NCBI. Phylogenetic analyses were conducted using the MEGA4 software [19]. Construction of a phylogenetic tree was performed using the ClustalW multiple alignment algorithm and Neighbor-Joining method with 1000 bootstrap replicates.

### Database accession numbers

The complete genome sequence of IHNV 220-90 strain has been deposited in GenBank with the accession no. GQ413939. The accession numbers of other viral sequences used for sequence comparison and phylogenetic analysis are listed (see additional File 1: Information about the infectious hematopoietic necrosis virus (IHNV) isolates used in this study for comparison and phylogenetic analysis).

## Results

### The complete nucleotide sequence of 220-90

**Figure 1:**
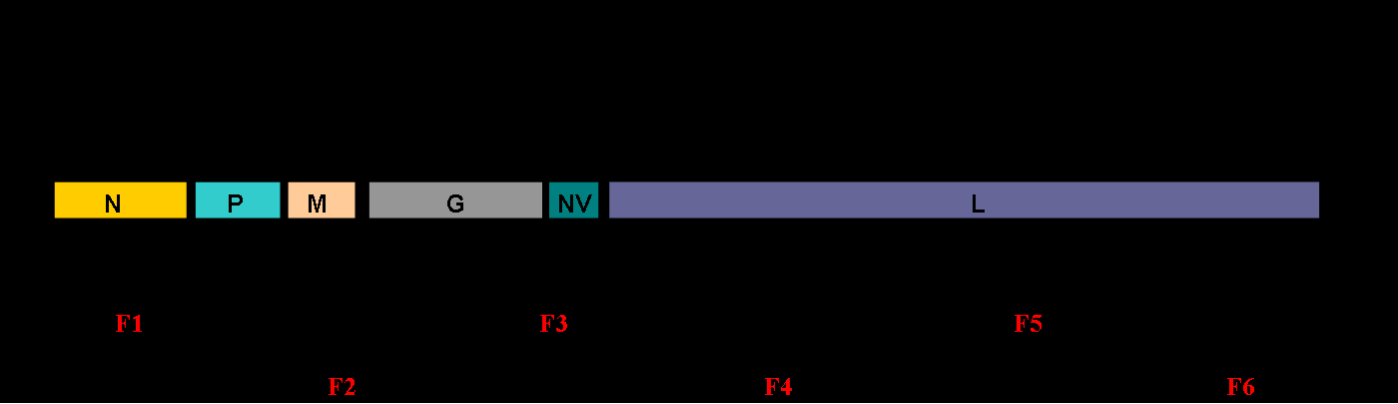
**Genetic map of the IHNV genome and cDNA clones used for sequence analysis**. The location and relative size of the IHNV ORFs are shown; the numbers indicate the starts and ends of the respective ORFs. Six cDNA fragments (F1 to F6) were synthesized from the genomic RNA by RT-PCR. The primers used for RT-PCR fragments are shown at the end of each fragment. The RNA genome is 11,133 nucleotides long and contains a leader (L) and trailer (T) sequences at its 3'-end and 5'-end, respectively. The coding regions of N, P, M, G, NV and L genes are separated by intergenic sequences, which have gene-start and gene-end signals.

The entire genome of IHNV 220-90 strain was amplified as six overlapping cDNA fragments that were cloned, and the DNA was sequenced (Fig. 1). The complete genome sequence of 220-90 comprises 11,133 nucleotides (nts) and contains six genes that encode the nucleocapsid (N) protein, the phosphoprotein (P), the matrix protein (M), the glycoprotein (G), the non-virion (NV) protein, and the large (L) protein (Fig. 1), The gene order is 3'-N-P-M-G-NV-L-5', like other novirhabdoviruses. The genomic features and predicted proteins of 220-90 are given in Table 2. All the genes are separated by untranslated sequences that are called gene junctions. The untranslated regions at the 3' and 5' ends are called the 'leader' and 'trailer', respectively. An additional uracil nucleotide was found at the end of the 5'-trailer region, which was not reported before in other IHNV strains.

**Table 2:** Genomic features and predicted proteins of the IHNV strain 220-90

S. No	Gene	Start	End	5'UTR	ORF	3'UTR	Total Length^a^	Protein Size (aa)	MW^b^kDa
1.	Leader	1	60				60		

2.	N	63	1430	112	1176	80	1638	391	42.3

3.	P	1433	2199	33	693	41	767	230	26.0

4.	M	2202	2945	53	588	103	744	195	22.0

5.	G	2948	4567	51	1527	42	1620	508	56.6

6.	NV	4570	4938	26	336	7	369	111	13.2

7.	L	4941	11031	76	5961	54	6091	1986	225.0

8.	Trailer	11032	11133				102		

^a ^Total length of a gene including 5'UTR, ORF and 3'UTR

^b ^Predicted molecular weight of proteins in kilodaltons (kDa)

### ORF 1 or Nucleocapsid (N) protein gene

The first ORF, extending from nts 175-1350, contains 391 residues and it encodes nucleoprotein (N) with a deduced molecular mass of 42 kDa. The N gene starts with the conserved sequence (CGUG) and has the putative polyadenylation signal (UCUUUUUUU). The 5'-untranslated region of 174 nts is followed by the first AUG codon of the 1176 nts open reading frame (ORF). Comparison of the published IHNV nucleoprotein sequences with IHNV 220-90 shows that it is 98% identical to the 193-110, HO-7 and LR-80 isolates (Table 3). The ORF 1 has 5' untranslated region of 112 nts (from putative gene start to AUG) and 3' untranslated region of 80 nts (from stop codon to the gene end).

**Table 3:** Percent (%) nucleotide or deduced amino acid identity of the IHNV strain 220-90 with other IHNV strains and Novirhabdoviruses^a^

IHNV Strains	3' Leader^¥^	N	P	M	G	NV	L	5' Trailer^¥^
193-110	-	**98**	-	-	**97**	96	-	-

332	-	-	-	-	**97**	-	-	-

Auke77	-	-	-	-	**97**	-	-	-

Carson-89	-	**96**	-	-	**97**	-	-	-

Col-80	-	95	-	-	**96**	-	-	-

Col-85	-	95	-	-	**96**	-	-	-

Cro/05	-	-	-	-	**97**	96	-	-

CST-82	-	**97**	-	-	**97**	96	-	-

G4	-	-	-	-	**96**	-	-	-

IHNV-PRT	-	93	95	98	**95**	95	-	-

FR0031	-	-	-	-	**96**	-	-	-

FF030-91	-	-	-	-	**96**	-	-	-

Fs42/95	-	-	-	-	**97**	97	-	-

Fs62/95	-	-	-	-	**97**	-	-	-

FsK/88	-	-	-	-	**97**	-	-	-

FsVi100/96	-	-	-	-	**97**	-	-	-

HO-7	-	**98**	-	-	**97**	97	-	-

HV7601	-	-	-	**98**	**97**	97	-	-

J04321	-	95	-	-	-	-	-	-

LB91KI	-	**96**	-	-	-	-	-	-

LR-73	-	95	-	-	**97**	96	-	-

LR-80	-	**98**	-	-	**97**	97	-	-

LWS-87	-	**96**	-	-	**97**	-	-	-

WRAC	96	**97**	**98**	**98**	**97**	96	98	96

RB-76	-	**96**	-	-	**97**	-	-	-

RB-1	-	**96**	-	-	97	97	-	-

RtUi02	-	**-**	-	-	94	-	-	-

SRCV	-	95	-	-	**96**	-	-	-

Strain K	-	**97**	**97**	**98**	**97**	97	98	-

X89213	96	**97**	**97**	**98**	**97**	97	98	95

**HIRRV**	64	62	65	74	74	53	84	71

**SHRV**	44	42	30	35	39	10	58	36

**VHSV**	41	40	35	36	38	10	60	29

^a ^more than 95% identities are shown in bold letters

^¥ ^only nucleotide sequences were used for analysis

Viruses belonging to *Novirhabdovirus *genus are in bold letters

HIRRV, Hirame rhabdovirus; SHRV, Snakehead rhabdovirus; VHSV, Viral hemorrhagic septicemia virus

### ORF 2 or Phosphoprotein (P) gene

The P gene of 220-90 is 767 nts long and encodes a protein of 230 amino acids (aa) with a predicted MW of 26.0 kDa (Table 2). The predicted P protein contains 6 serine, 5 threonine and 1 tyrosine residues, identified as possible phosphorylation sites using NetPhos 2.0 server http://www.cbs.dtu.dk/. Among novirhabdoviruses, the IHNV-P protein has an amino acid sequence identity of 35% with viral hemorrhagic septicemia virus (VHSV), 65% with Hirame rhabdovirus (HIRRV), and 30% with snakehead rhabdovirus (SHRV) (Table 3).

### ORF 3 or Matrix (M) gene

The M gene of 220-90 is 744 nts long and encodes an M protein of 195 aa residues with a predicted MW of 22.0 kDa (Table 2). Among novirhabdoviruses, the M protein has an amino acid sequence identity of 36% with VHSV, 74% with HIRRV, 35% with SHRV (Table 3). A 5'-untranslated region of 53 nts is followed by an ORF and succeeded by 103 nts 3' UTR.

### ORF 4 or glycoprotein (G) gene

The gene for the G protein is located between 2948 and 4567 nts from the 3'-end of the viral genome. A 3' UTR of 51 nts is followed by an ORF (nts 1524) that encodes a polypeptide of 508 aa residues, with a calculated MW of 56.6 kDa, and succeeded by 42 nts 3' UTR. The predicted G protein contains 20 serine, 6 threonine and 6 tyrosine residues, identified as possible phosphorylation sites using NetPhos 2.0 server http://www.cbs.dtu.dk/. Four putative N-glycosylation sites were identified at amino acids 56-59 (NASQ), 400-403 (NNTT), 401-404 (NTTI) and 438-441(NETD) and one O-glycosylation were identified at amino acid position 492. We compared the G protein of 28 IHNV strains from different parts of the world. The regions between amino acid positions 32-52, 131-204, 289-369, 380-416 are highly conserved. The regions between amino acids 247-257 and 269-276 have a greater genetic diversity than any other part of the G protein. The IHNV glycoprotein has the following domains: signal peptide at N-terminal (1-20aa), ectodomain (21-459aa), transmembrane domain (460-482 aa) and endodomain (483-508 aa), which were predicted by SignalP server http://www.cbs.dtu.dk/services/SignalP/.

### ORF 5 or Non-virion (NV) protein gene

The NV protein gene is located between 4570 and 4938 nts from the 3'-end of the viral genome. It encodes a polypeptide of 111 aa residues, with a calculated molecular mass of 13.2 kDa. The predicted NV protein contains 1 serine, 2 threonine and 1 tyrosine residues, identified as possible phosphorylation sites using NetPhos 2.0 server http://www.cbs.dtu.dk/. The function of NV protein is not clearly known. NV is a non-structural protein of novirhabdoviruses, which could be detected only in the infected cells [20].

### ORF 6 or Polymerase (L) gene

ORF 6 encodes the largest protein, the polymerase, which starts at position 5017 and ends at position 10977. It encodes a polypeptide of 1986 aa residues, with a deduced molecular mass of 225.0 kDa. The L protein contains 67 serine, 38 threonine and 9 tyrosine residues as possible phosphorylation sites. The predicted RNA-dependent RNA polymerase (RdRp) domain is situated between residues 18 and 1159. The deduced L protein of IHNV exhibits 60%, 84%, and 58% identities with VHSV, HIRRV and SHRV, respectively (Table 3).

### The Genomic termini and untranslated sequences

**Figure 2:**
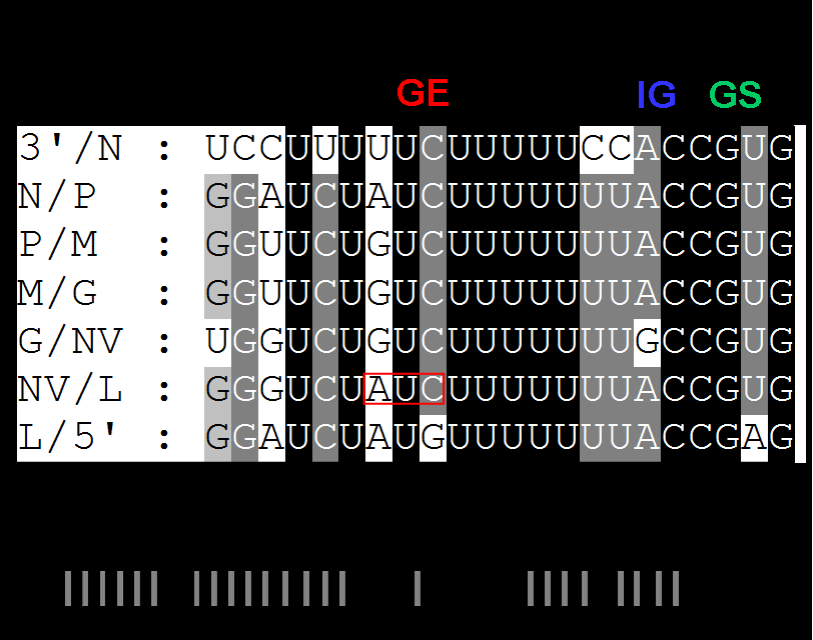
**Analysis of the gene junctions and complementarities in the IHNV genome**. **A**) Seven identified gene junctions of IHNV in the negative-sense of the genomic RNA are shown. 3'/N, junction of 3'-leader and nucleocapsid gene; N/P, junction of nucleocapsid and phosphoprotein gene; P/M, junction of phosphoprotein and matrix gene; M/G, junction of matrix and glycoprotein gene; G/NV, junction of glycoprotein and non-virion gene; NV/L, junction of non-virion and polymerase gene; L/5'-, junction of polymerase gene and 5' trailer. GE = Gene end; IG = Intergenic di-nucleotide; GS = Gene start. The stop codon of NV ORF is merged with gene end sequence and is shown in red box. **B) **Complementarities of the 3'- and 5'-ends of the IHNV genome. The first 15 of the 16 nucleotides at the 3'-end are complementary to the 5'-end nucleotides of genomic RNA.

Rhabdoviruses have conserved untranslated regions between open reading frames for optimal translation of viral proteins [21]. These sequences consist of a putative transcription stop/polyadenylation motif (UCURUCU^7^) which signals reiterative copying of the U sequences to generate poly (A) tail to the mRNA. This sequence is followed by an intergenic di-nucleotide AC or GC which are not transcribed, and a putative transcription start signal, CGUG (Fig. 2A). The gene junctions of different novirhabdoviruses are shown in Table 4.

**Table 4:** Comparisons of the gene junctions of the IHNV genome with other Novirhabdoviruses

Type Species		Gene Junctions	
	**N/P**	**P/M**	**M/G**

**IHNV**	**UCU **A**UCUUUUUUU **AC **CGUG**AUAUCACG	**UCU**G**UCUUUUUUU **AC **CGUG**CGUUCACA	**UCU**G**UCUUUUUUU **AC **CGUG**AAAACACG

**SHRV**	**UCU**A**UCUUUUUUU **GC **CGUG**CUCUCACG	**UCU**G**UCUUUUUUU **AC**CGUG**CUCUCACG	**UCU**G**UCUUUUUUU **AC **CGUG**CUCUCACG

**VHSV**	**UCU**A**UCUUUUUUU **GC **CGUG**CUAAUAUU	**UCU**A**UCUUUUUUU **GC **CGUG**CUGACAAG	**UCU**A**UCUUUUUUU **AC **CGUG**UAAACACA

**HIRRV**	**UCU**A**UCUUUUUUU **AC **CGUG**CAAACACA	**UCU**A**UCUUUUUUU **AC **CGUG**CAAUCACA	**UCU**A**UCUUUUUUU **AC **CGUG**UAAACACA

	**G/NV**	**NV/L**	

**IHNV**	**UCU**G**UCUUUUUUU **GC **CGUG**UAAACACG	**UCU**A**UCUUUUUUU **AC **CGUG**AAAACACG	

**SHRV**	**UCU**G**UCUUUUUUU**U GC **CGUG**AUAUCACG	**UCU**A**UCUUUUUUU **GC **CGUG**CAUUACACG	

**VHSV**	**UCU**A**UCUUUUUUU **AC **CGUG**GAAAUACU	**UCU**A**UCUUUUUUU **AC **CGAG**AAAACAAC	

**HIRRV**	**UCU**A**UCUUUUUUU **GC **CGUG**UAUACAGA	**UCU**A**UCUUUUUUU **AC **CGUG**AACACACG	

The gene junctions shown here are negative-sense RNA sequences of respective viruses. IHNV, Infectious hematopoietic necrosis virus; SHRV, Snakehead rhabdovirus; VHSV, Viral hemorrhagic septicemia virus; HIRRV, Hirame rhabdovirus

The untranslated region of 3' leader and 5' trailer are 60 nts and 102 nts in length, respectively. The 3' leader of 220-90 is 63% A/T rich, whereas 5' trailer is 60% A/T rich. Like other rhabdoviruses, the genomic termini of IHNV 3'-terminal nucleotides exhibit complementarities to the nucleotides of 5'-terminus of the genomic RNA (Fig. 2B). The complementary nature of genomic termini involves the formation of a panhandle structure, which is important for replication of rhabdoviruses.

### Homology and phylogenetic analysis

Phylogenetic trees were generated from the nucleotide sequences of the ORFs and of the complete genome. The complete genome and gene proteins of IHNV were also compared with different members of novirhabdoviruses and the results are shown in Tables 3 and 4. Among novirhabdoviruses, HIRRV is closely related to IHNV and has an identity of 72%. Comparison of the UTRs and protein coding sequences of 220-90 strain with novirhabdoviruses shows that non-virion protein is highly variable than any other region of the genome (Table 3). The 3'- and 5'- UTRs are more conserved among *Rhabdoviridae *family members than protein coding genes (data not shown). The complete genome comparison of 220-90 with other two available sequences of IHNV strains reveals 96% identity with WRAC, and 95% with strain K (X89213).

**Figure 3:**
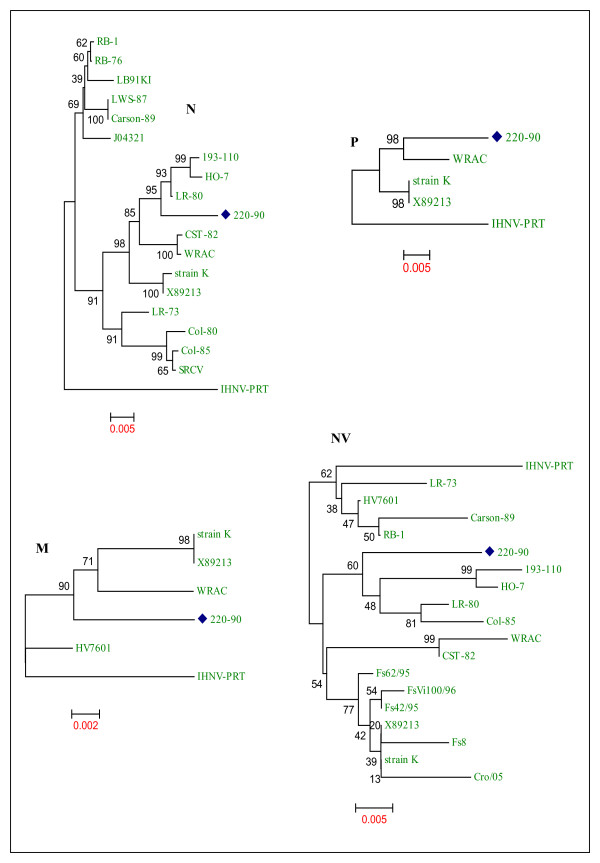
**Phylogenetic tree analysis of sequences of nucleocapsid (N), matrix (M), phosphoprotein (P), and non-virion protein (NV) of various IHNV strains**. Information about the IHNV strains used in this analysis is described in additional file 1. IHNV 220-90 strain is marked with blue diamond. Phylogenetic tree analysis was conducted by neighbor-joining method using 1000 bootstrap replications. The scale at the bottom indicates the number of substitution events and bootstrap confidence values are shown at branch nodes.

**Figure 4:**
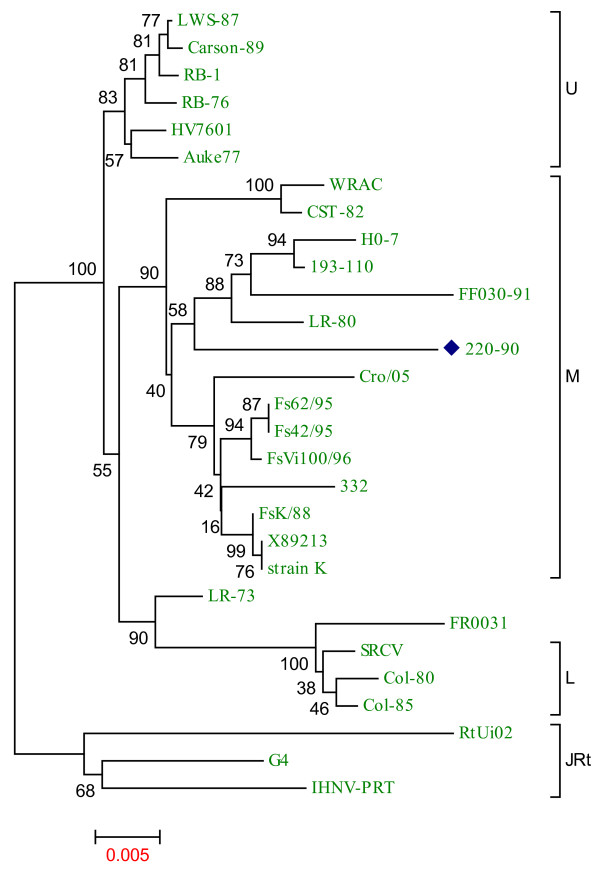
**Phylogenetic relationship of the full-length glycoprotein (G) sequences of 28 IHNV strains with IHNV 220-90**. Genogroups are depicted by vertical lines, as described by [10]. Brackets indicate the three major genogroups, U, M and L. IHNV 220-90 (blue diamond) is grouped under M genogroup. Data of virus isolates used here are available in additional file 1. Phylogenetic tree analysis was conducted by neighbor-joining method using 1000 bootstrap replications. The scale at the bottom indicates the number of substitution events and bootstrap confidence values are shown at branch nodes.

The phylogenetic tree analysis of sequences of nucleocapsid (N), matrix (M), phosphoprotein (P), and non-virion protein (NV) of various IHNV strains are shown in Fig. 3. Phylogenetic analysis of the N gene shows clustering of 220-90 with HO-7, 193-110 and LR-80 and maintains 98% identity with those strains. Among the available sequences, WRAC strain exhibits very close identity (98%) with 220-90 for both P and M genes. All the strains display 98% identity with the 220-90 M gene, which demonstrates the highly conserved nature of M gene. When the NV genes were compared, 220-90 strain shows 95-97% identity with other IHNV strains. Previously, the North American IHNV isolates were genogrouped as U, M and L based on glycoprotein sequences [10]. Phylogenetic tree of the G genes displays that 220-90 strain belongs to the M genogroup (Fig. 4).

## Discussion

A virulent IHNV strain 220-90 was isolated from the hatchery-reared juvenile rainbow trout during 90's in the Hagerman Valley, Idaho, USA [8]. IHNV is endemic throughout the Pacific Northwest region of North America, with range extending from Alaska to northern California along the Pacific coast and inland to Idaho. It causes systemic disease predominantly in both wild and cultured salmon and trout [1,2,10]. The disease typically occurs in rainbow trout fry maintained in the multiple outdoor rearing units of rainbow trout farm facilities [8,12].

To date, the complete genome sequences are available for only two IHNV strains [13,14]. Previously, only the G protein gene sequence for 220-90 strain was determined (GenBank accession no. DQ164101). Comparison of the G gene sequence of 220-90 isolate with the published sequence of the same shows nine nucleotide changes, which results in 7 aa changes. This may be due to different passage number of the virus in cell culture. To fully understand the molecular characteristics of a virulent IHNV, we determined the complete nucleotide sequence of 220-90 strain. The genome is 11,133 nts long and the gene organization (N, P, M, G, NV and L) is similar to all members of the *Novirhabdovirus *genus. The termini of the viral genome have conserved sequences at the 3'-end (CAUAU) and at the 5'-end (GUAUA) as other members of *Novirhabdovirus *genus. Out of first 16 nucleotides of the 3'-terminus, 15 nucleotides are complementary to 5'-terminus of the genome (Fig. 2B), which forms the panhandle structure that may be involved in replication [22]. The length of the 3'-leader of 220-90 is 60 nts, which is similar to HIRRV but slightly shorter than VHSV and SHRV (53 nts). IHNV has the second longest 5' trailer (120 nts) than other novirhabdoviruses, such as VHSV (116 nts), SHRV (42 nts), and HIRRV (73 nts). Even though the length of 3'-leader is consistent between the members of genus *Novirhabdovirus*, the length of the 5'-trailer is highly variable (from 42nt to 116nt). It is possible that the difference in the length of trailer sequences may have some functional significance, which remains to be seen.

**Figure 5:**
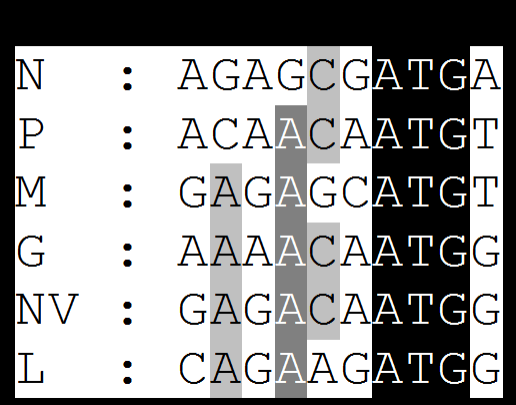
**Kozak sequence context of each gene of IHNV 220-90**. Sequences shown here are positive-sense anti-genome. * Conserved adenosine (A) at position -3. ** Start codon (ATG)

All the genes of VHSV start with a conserved gene start sequence (-CGUG-) like other novirhabdoviruses, followed by an ORF and conserved gene-end sequence (A/GUCUAU/ACU^7^). All the genes end with 7 uracil (U) residues, which are polyadenylation signal for polymerase when it transcribes a gene. Polymerase adds poly (A) by stuttering mechanism [23]. After this poly (A) signal, there are two conserved intergenic di-nucleotides (G/AC), which are untranscribed and act as spacers between two genes. Polymerase skips these two nucleotides to next gene start sequence and starts transcribing next gene [23]. Transcription of rhabdovirus mRNAs is regulated by cis-acting signals located within the 3' leader region and untranslated region between each gene ORF [23-26]. In case of NV, the stop codon of NV gene is merged with gene-end sequences (Fig. 2A). Transcription of rhabdovirus mRNAs is regulated by cis-acting signals located within the 3' leader region and untranslated region between each gene ORF [23-26]. The Kozak context for each gene was compared, as shown in Fig. 5. At position -3, all the genes have adenosine (A) nucleotide, except the ORF of N gene.

We observed that aa residues between 1-22, 106-150 and 206-268 are highly conserved in the N protein, whereas residues 30-31, 41-43, 177-181, 203-205 and C-terminal region from residue 312 are variable. Phylogenetic analysis of the N protein shows grouping of 220-90 with LR-80, HO-7 and 193-110 strains, with an identity of 98%. Phylogenetic tree of the P protein shows clustering of 220-90 with WRAC strain, having an identity of 98%. The matrix (M) protein is an important structural component of virion, forming a layer between the glycoprotein containing outer membrane and the nucleocapsid core. Matrix protein of IHNV is highly conserved (Table 3). IHNV strains used in this study exhibit very close (98%) identity with 220-90. In phylogenetic analysis of M protein, WRAC and strain K, which is the same strain as Kinkelin from France (X89213), form a cluster that exhibit 99-100% identity with each other, and 98% identity with 220-90. Matrix protein of rhabdovirus is involved in viral assembly, condensation of nucleocapsid, formation of bullet-shaped virion [27,28] and induces apoptosis by shutdown of host cell machinery in infected cells [29,30]. Because it is highly essential for assembly and release of virion, the matrix protein maintains highest homology among IHNV along with the polymerase protein.

The non-virion protein (NV) of 220-90 shows identity of 95-97% with other IHNV strains. The NV protein of IHNV is conserved than counterpart of VHSV, which showed high genetic diversity [17]. It was demonstrated that NV-knockout IHNV replicated very slowly in cell culture and was non-pathogenic in fish [31]. On the contrary, NV-knockout SHRV replicated very well as wild-type virus and it was shown that NV protein of SHRV is not essential for pathogenesis [32]. These studies suggested that each species of *Novirhabdovirus *genus has its own characteristics and one can not ignore the importance NV in pathogenesis. The conserved nature of NV and its importance for growth and pathogenesis suggests that NV is highly essential for IHNV. All the available L sequences for IHNV strains show highest conservation (98%) as that of matrix protein (Table 3). The L protein is packaged into the virus particle and is involved in both transcription and replication [23].

Genomic comparison of IHNV strains isolated from various marine species from different parts of the world sheds light on the correlation of genetic sequences with viral tropism and pathogenicity. The glycoprotein (G) is believed to be involved in virulence and tropism because it's involvement in viral attachment and cell entry [33]. Comparison of glycoproteins of various IHNV strains has shown long blocks of conserved region (data not shown). The regions between residues 8-22; 32-52; 131-214; 289-369; and 380-416 are highly conserved and the rest is showing genetic variations, which are scattered all over the protein. The major neutralizing epitopes have been mapped to two antigenic sites for IHNV, at amino acid residues 230-231 and 272-276 [34,35]. In this analysis, we found no amino acid substitutions at positions 230-231 among 28 strains compared. On the other hand, residues 270-276 are highly variable, which supports earlier findings [34,35], and suggests the involvement of this site in antigenic variation and virulence.

A wide sequence analysis of mid-G region (303 nts) within the glycoprotein gene of 323 North American IHNV isolates revealed a maximum nucleotide diversity of 8.6%, indicating low genetic diversity overall for this virus [10]. The North American IHNV isolates, genogrouped as U, M and L by phylogenetic analysis, vary in topography and geographical range [10]. The phylogenetic analysis of the glycoprotein of 220-90 (Fig. 4) shows clustering with LR-80, FF030-91, 193-110 and HO-7 strains, which exhibits that 220-90 belongs to the M genogroup.

## Competing interests

The authors declare that they have no competing interests.

## Authors' contributions

VNV and SEL conceived the study. AA planned the experimental design and carried out cloning and sequencing. AA drafted the manuscript. All authors critically reviewed and approved the final manuscript.

## Supplementary Material

Additional file 1 Information about the infectious hematopoietic necrosis virus (IHNV) isolates used in this study for comparison and phylogenetic analysis Click here for file
