# Supplementary material for: Molecular characterization of the virulent infectious hematopoietic necrosis virus (IHNV) strain 220-90
Source: Virol J. 2010 Jan 19;7:10. doi: 10.1186/1743-422X-7-10 (PMC2820013; doi:10.1186/1743-422X-7-10)
Supplement: Additional file 1 — Information about the infectious hematopoietic necrosis virus (IHNV) isolates used in this study for comparison and phylogenetic analysis [file 1743-422X-7-10-S1.DOC]

**Additional file 1: Information about the infectious hematopoietic necrosis virus (IHNV) isolates used in this study for comparison and phylogenetic analysis**

| **S. No** | **Strain** | **Country** | **Host** | **GenBank no.** |
| --- | --- | --- | --- | --- |
| **N protein** | | | | |
|  | SRCV | USA | Chinook salmon | AY442517 |
|  | RB-76 | USA | Steelhead trout | AY442516 |
|  | RB-1 | USA | steelhead trout | U50402 |
|  | IHNV-PRT | Korea | rainbow trout | AY673683 |
|  | LWS-87 | USA | Chinook Salmon | AY4425157 |
|  | LR-80 | USA | Chinook Salmon | AY442514 |
|  | LR-73 | USA | Chinook Salmon | AY442513 |
|  | HO-7 | USA | Steelhead × Rainbow Juvenile | AY442512 |
|  | CST-82 | USA | rainbow trout | AY442511 |
|  | Col-85 | USA | Chinook | AY442510 |
|  | Col-80 | USA | Steelhead | AY442509 |
|  | Carson-89 | USA | Chinook | AY442508 |
|  | 193-110 | USA | Rainbow | AY442507 |
|  | LB91KI | USA | Salmon | AY438975 |
|  | Strain K | France |  | X73872 |
| **P protein** | | | | |
|  | IHNV-PRT | Korea | rainbow trout | AY673685 |
|  | Strain K | France |  | X73872 |
| **M protein** | | | | |
|  | HV7601 | Japan |  | AB231685 |
|  | IHNV-PRT | Korea | rainbow trout | AY673686 |
|  | Strain K | France |  | X73872 |
| **G protein** | | | | |
|  | IHNV-PRT | Korea | rainbow trout | AY673684 |
|  | LR-73 | USA | Chinook Salmon | L40877 |
|  | LR-80 | USA | Chinook Salmon | L40878 |
|  | Carson-89 | USA | Chinook | L40872 |
|  | RB-1 | USA | steelhead trout | U50401 |
|  | RB-76 | USA | Steelhead trout | L40880 |
|  | 193-110 | USA | Rainbow | L40871 |
|  | HO-7 | USA | Steelhead × Rainbow Juvenile | L40876 |
|  | LWS-87 | USA | Chinook Salmon | L40879 |
|  | HV7601 | Japan |  | AB231686 |
|  | Auke77 | USA | sockeye salmon | DQ164099 |
|  | CST-82 | USA | rainbow trout | L40875 |
|  | FF030-91 | USA | rainbow trout | DQ164103 |
|  | Cro/05 | Croatia | rainbow trout | EU219616 |
|  | Fs62/95 | Germany |  | AY331664 |
|  | Fs42/95 | Germany |  | AY331663 |
|  | FsVi100/96 | Germany |  | AY331666 |
|  | 332 | Germany |  | AY331657 |
|  | FsK/88 | Germany |  | AY331665 |
|  | FR0031 | USA | Chinook Salmon | DQ164102 |
|  | SRCV | USA | Chinook salmon | L40881 |
|  | Col-80 | USA | Chinook | L40873 |
|  | Col-85 | USA | Steelhead | L40874 |
|  | RtUi02 | Korea | rainbow trout | AB288207 |
|  | G4 | Japan | rainbow trout | AF244128 |
|  | Strain K | France |  | X73872 |
| **NV Protein** | | | | |
|  | IHNV-PRT | Korea | rainbow trout | AY673687 |
|  | LR-73 | USA | Chinook Salmon | L40877 |
|  | LR-80 | USA | Chinook Salmon | L40878 |
|  | Carson-89 | USA | Chinook | L40872 |
|  | RB-1 | USA | steelhead trout | U47846 |
|  | RB-76 | USA | Steelhead trout | L40880 |
|  | 193-110 | USA | Rainbow | L40871 |
|  | HO-7 | USA | Steelhead × Rainbow Juvenile | L40876 |
|  | fs8 | Germany |  | AY780893 |
|  | LWS-87 | USA | Chinook Salmon | L40879 |
|  | HV7601 | Japan |  | AB231659 |
|  | CST-82 | USA | rainbow trout | L40875 |
|  | Cro/05 | Croatia | rainbow trout | EU219617 |
|  | Fs42/95 | Germany |  | AY780896 |
|  | Fs62/95 | Germany |  | AY780897 |
|  | FsVi100/96 | Germany |  | AY780898 |
|  | SRCV | USA | Chinook salmon | L40881 |
|  | Col-85 | USA | Steelhead | L40874 |
|  | Strain K | France |  | X73872 |
| **Complete genome** | | | | |
|  | WRAC | USA | Chinook salmon | L40883 |
|  |  | France | rainbow trout | X89213 |
| **Rhabdoviruses Complete Genome** | | | | |
|  | **Rhabdovirus** | | | **GenBank no.** |
|  | Bovine ephemeral fever virus (BEFV) | | | NC_002526 |
|  | European bat lyssavirus (Bat) | | | NC_009527 |
|  | Northern cereal mosaic virus (Cereal) | | | NC_002251 |
|  | Lettuce necrotic yellows virus (Lettuce) | | | NC_007642 |
|  | Maize Fine streak virus | | | NC_005974 |
|  | Maize mosaic virus (MMV) | | | NC_005975 |
|  | Mokola virus | | | NC_006429 |
|  | Orchid fleck virus (OFV) | | | NC_009609 |
|  | Rabies virus | | | NC_001542 |
|  | Siniperca chuatsi rhabdovirus | | | NC_008514 |
|  | Spring viremia of carp virus (SVC) | | | NC_002803 |
|  | Sonchus yellow net virus (SYN) | | | NC_001615 |
|  | Taro vein chlorosis virus (Taro) | | | NC_006942  NC_006942  NC_006942 |
|  | Tupaia rhabdovirus | | | NC_007020 |
|  | Vesicular stomatitis virus (VSV) | | | NC_001560 |
|  | Viral hemorrhagic septicemia virus | | | GQ385941 |
|  | Hirame rhabdovirus (HIRRV) | | | NC_005093 |
|  | Snakehead rhabdovirus (SHRV) | | | NC_000903 |

This table shows the name of IHNV strains, countries or places from which strains were isolated and their GenBank accession numbers. These data were taken from National Center for Biotechnology Information (NCBI) website.
